# Exploration the Significance of a Novel Immune-Related Gene Signature in Prognosis and Immune Microenvironment of Breast Cancer

**DOI:** 10.3389/fonc.2020.01211

**Published:** 2020-07-24

**Authors:** Yajie Zhao, Chunrui Pu, Zhenzhen Liu

**Affiliations:** Department of Breast, Affiliated Cancer Hospital of Zhengzhou University & Henan Cancer Hospital, Zhengzhou, China

**Keywords:** bioinformatics, immune, breast cancer, The Cancer Genome Atlas (TCGA), Gene Expression Omnibus (GEO)

## Abstract

This study was designed to identify an immune-related gene signature (IRGS) associated with breast cancer (BC) patient outcomes. Transcriptomic data from 1411 BC patients in the TCGA and GEO databases were used to identify differentially expressed immune-related genes (DEIGs) when comparing BC tumor and normal tissue samples. We were able to construct a 27-gene IRGS that was able to effectively separate BC patients into high- and low-risk groups that corresponded to significant differences in overall and recurrence-free survival (OS and RFS, respectively). Besides, the relevance of this signature to immune response and immune cell infiltration of BC tumors was evaluated. These high- and low-risk BC patients were found to exhibit significantly different immune responses and functional enrichment. We also identified patients in the high-risk group exhibited significantly reduced immune cell infiltration of tumors relative to low-risk patients. Together, the results of this analysis offer a novel overview of the immune microenvironment within BC tumors and highlight key immunological genes associated with patient survival outcomes.

## Introduction

Breast cancer (BC) remains the most common form of cancer globally (1). There have been numerous diagnostic and therapeutic improvements over the past 30 years that have facilitated a ~33% reduction in BC mortality (2). At present, however, there has been relatively little focus on immunotherapeutic BC treatment owing in part to the fact that these tumors typically exhibit low-to-moderate levels of immunogenicity (3). While immunotherapies for the treatment of melanoma, non-small cell lung cancer (NSCLC) and other tumors have become increasingly advanced, the study of such approaches in the context of BC is still in its infancy.

Preclinical studies in recent years have clearly demonstrated that both local inflammation and the immune landscape of the tumor stromal compartment can influence tumor development and progression (4, 5). This has led to an increasing focus on the identification of specific immune-related genes or gene signatures that can offer insight into BC progression and/or therapeutic susceptibility. Immunological biomarkers have been successfully identified in a number of different oncogenic contexts (6–9). For example, one recent study (8) was able to develop a signature of 27 immune-related genes associated with the overall survival (OS) of head and neck squamous cell carcinoma patients. Multiple immunological parameters have been associated with BC patient outcomes, further efforts to identify prognostic immunological biomarkers associated with BC tumor microenvironmental status are thus warranted as they have the potential to guide patient treatment and to aid in the identification of novel immunotherapeutic interventions.

The tumor microenvironment (TME) is composed of the local stromal and immune cells which interact with and/or infiltrate a given tumor (10). Altered immune responses within the TME have been shown to be closely linked to tumor progression, with immune dysfunction often being supportive of tumor proliferation, migration, and metastasis. While in some cases immune cells are able to detect and destroy tumor cells, in many cases these tumors develop mechanisms that allow them to evade immunological detection. The specific composition of the immunological component of the BC TME is, at present, poorly understood, as few studies have conducted a thorough examination of the dynamics of immune cell infiltration into BC tumors or the plasticity of their responses therein owing to the difficulties inherent in such analyses.

In the present study, we therefore conducted a computational analysis wherein we leveraged transcriptomic data from BC patient samples in order to generate an immunological gene signature associated with patient survival outcomes. We then systematically validated the prognostic relevance of this gene signature and assessed its relationship to immune responses in BC tumors. Together, our results highlight a novel overview of the immune microenvironment within BC tumors and highlight key immunological pathways associated with patient survival outcomes.

## Materials and Methods

### Patients' Samples

We retrospectively analyzed publically available transcriptomic and clinical data from 1411 total BC patients, with training data being derived from The Cancer Genome Atlas (TCGA; *n* = 1,222, 113 normal breast samples and 1109 BC samples) and separate gene expression omnibus (GEO) microarray datasets (GSE42568, 104 BC samples; GSE7390, 198 BC samples) being used for validation. The GSE42568 data were collected using the GPL570 platform (Affymetrix Human Genome U133 Plus 2.0 Array), while the GSE7390 data were collected using the GPL96 platform (Affymetrix Human Genome U133A Array). Batch effects were eliminated using the “combat” function in the “sva” package. No ethical oversight was required for this study, as all data were derived from publically accessible sources.

### Identification of Differently Expression Immune-Related Genes

We utilized the “limma” package in order to normalize gene expression data and to identify differentially expressed (DE) genes when comparing BC and normal breast tissue samples in the TCGA database (false discovery rate < 0.05 and |log_2_ fold change| > 1). We then utilized lists of immune-related genes (*n* = 2,404) from the Immunology Database and Analysis Portal (ImmPort) (https://immport.niaid.nih.gov), in order to identified DE immune-related genes (DEIGs) within this dataset.

### Development and Validation of a DEIG-Based Prognostic Gene Signature

We utilized the “survival” R package (bioconductor.org/packages/survivalr/) in order to identify DEIGs associated with survival in our TCGA BC patient cohort through univariate Cox analyses, with a log-rank *p* < 0.05 as the selection threshold cutoff. We next utilized survival-related DEIGs in order to construct an immune-related gene signature (IRGS) that was predictive of BC patient survival. A least absolute shrinkage and selection operator (LASSO) regression approach was used to identify genes for incorporation into this signature. An IRGS-based prognostic risk score was then assigned based on the following formula: Risk score = expression of Gene 1 * β1 + expression of Gene 2 * β2 +…expression of Gene n * βn, with β corresponding to gene-specific regression coefficient values that were generated based on our TCGA training dataset. Median IRGS risk scores were then used to separate BC patients into low- and high-risk subgroups. The formula derived from the training dataset was then used to assess BC patients in our GEO validation cohort. The prognostic relevance of this IRGS was assessed using univariate analyses for BC patients in both the training and validation cohorts. We then evaluated the independent prognostic relevance of IRGS scores through multivariate analyses.

### Functional Annotation and Analysis

Principal components analysis (PCA) was used to assess gene expression patterns in grouped patients, while a gene set enrichment analysis (GSEA) approach was used to determine whether high- and low-risk BC patients exhibited distinct gene signatures. In total, two sets of genes (immune system process, M13664; immune response, M19817) derived from the Molecular Signatures Database v4.0 (http://www.broadinstitute.org/gsea/msigdb/index.jsp) were evaluated in this analysis. In addition, the cancer hallmarks and KEGG datasets from MSigDB were assessed through this same approach, with FDR < 0.05 and normalized enrichment score (NES) < 0.05 being used as significance cutoff criteria.

### Immune Infiltration Analysis

Immune and stromal cell populations in BC tumor samples were evaluated in an effort to establish the relationship between our IRGS signature and the BC TME. The Estimation of Stromal and Immune cells in Malignant Tumor tissues using the Expression data (ESTIMATE) approach was leveraged in order to estimate immune and stromal cell populations in BC tumor samples. In addition, the CIBERSORT algorithm was used for immune cell type-specific analyses. For TCGA BC data, both RNA-seq data and data regarding tumor infiltration frequencies by different immune cells including lymphocytes, monocytes, and neutrophils were available to support these analyses. Pathologic BC patient features in different risk groups were compared via Student's *t*-tests.

### Statistical Analysis

R (v3.6.3; http://www.Rproject.org) was used for statistical testing. The prognostic relevance of DEIGs was assessed via univariate Cox regression analyses, with hazard ratio (HR) values being used to determine the association between DEIG expression and patient risk (with *p* < 0.05 as a cutoff threshold). The “glmnet” package was used for LASSO regression analysis. The final IRGS was generated based on the sum of the expression levels of individual genes weighted according to individually determined regression coefficients (β). Kaplan-Meier curves and log-rank tests were used for prognostic evaluation. Factors independently associated with patient prognosis were identified through univariate and multivariate Cox regression analyses. The “survival ROC” package was employed for a time-dependent ROC curve analysis. Proportions of specific immune and stromal cells were estimated using ESTIMATE, and GraphPad Prism 8 (GraphPad Software Inc., CA, USA) was used for figure preparation. A *p*-value < 0.05 was the significance threshold.

## Result

### Identification of DEIGs From TCGA Dataset

**Figure 1:**
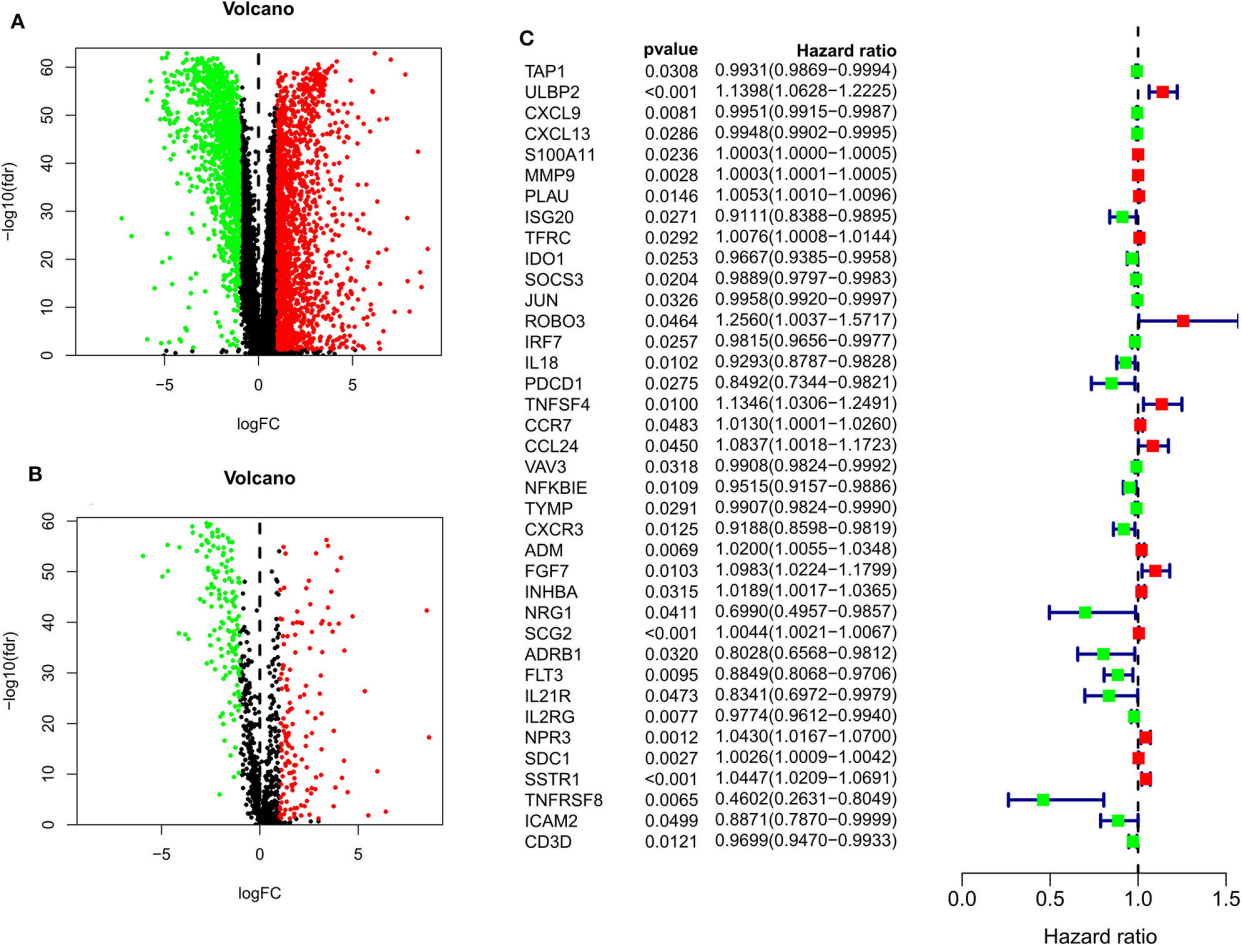
Volcano plots for expression of differentially expressed genes (Red represents the up-regulated genes, while green represents the downregulated genes) **(A)** Differentially expressed genes (Downregulated, *n* = 1,456; Upregulated, *n* = 1,832); **(B)** Differentially expressed immune-related genes (Downregulated, *n* = 146; Upregulated, *n* = 147). **(C)** Survival-associated DEIGs, a total of 38 survival-associated DEIGs showed in forest plot.

When comparing BC and normal breast tissues in the TCGA database, we were able to identify 3288 DE genes (1832 upregulated and 1456 downregulated) and 1293 DEIGs (1147 upregulated and 146 downregulated) (FDR < 0.05 and |log_2_ fold change| > 1). Data were arranged in volcano plots (Figure 1).

### Immune-Related Gene Signature (IRGS) Construction and Validation

**Figure 2:**
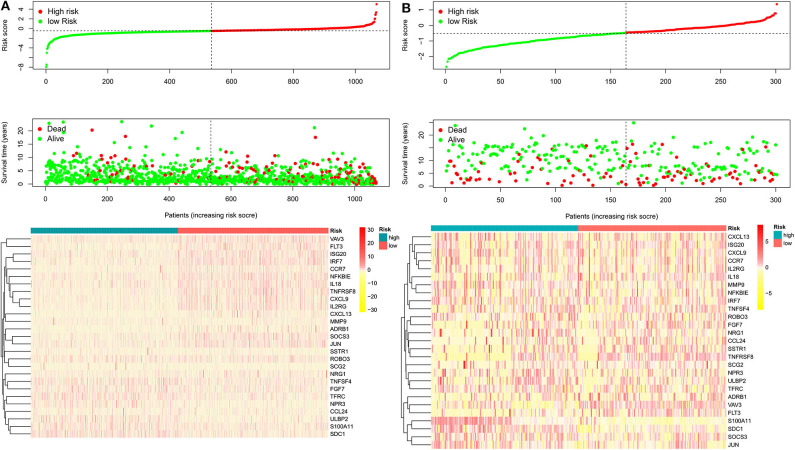
The heatmap and distribution of the 27 genes expression profiles in the high-risk and low-risk subgroups for the training cohort **(A)** and validation cohort **(B)**.

After patients lacking survival data were removed from our TCGA and GEO datasets, we compiled BC patient data for both a training cohort (*n* = 1,069) and a validation cohort (*n* = 301) (Table 1). We initially identified 38 DEIGs that were significantly associated with BC patient OS in the training cohort (Figure 1), and we then employed a LASSO Cox regression approach in order to identify, 27 of these genes for incorporation into the final IRGS (Table 2). The prognostic relevance of IRGS-derived risk scores was then evaluated in our training cohort, with BC patients being divided into high- and low-risk subsets based on the median risk score value within this group (−0.44899) (Figure 2).

**Table 1:** Clinical characteristics for training and validation cohort.

	**Training cohort**	**Validation cohort**
	**TCGA (*n* = 1,069)**	**GSE (*n* = 301)**
Age (year)	58.09 ± 12.93	50.71 ± 10.8
**T stage**		
T1	279 (26.1%)	136 (45.18%)
T2	617 (57.72%)	163 (54.15%)
T3	132 (12.35%)	2 (0.67%)
T4	38 (3.55%)	0 (0%)
Tx	3 (0.28%)	0 (0%)
**N stage**		
N0	507 (47.43%)	242 (80.4%)
N+	550 (51.45%)	59 (19.6%)
Nx	12 (1.12%)	0 (0%)
**M stage**		
M0	1036 (96.91%)	301 (100%)
M1	22 (2.06%)	0 (0%)
Mx	11 (1.03%)	0 (0%)

**Table 2:** 27-gene immune signature.

**Gene symbol**	**Gene ID**	**Description**	**Coefficient**
ULBP2	80328	UL16 binding protein 2	0.072825488
CXCL9	4283	Chemokine (C-X-C motif) ligand 9	−0.002404526
CXCL13	10563	Chemokine (C-X-C motif) ligand 13	−0.000428563
S100A11	6282	S100 Calcium Binding Protein A11	0.000205037
MMP9	4318	Mtrix metallopeptidase 9	0.000233893
ISG20	3669	Interferon-stimulated exonuclease gene 20	−0.006115365
TFRC	7037	Transferrin receptor	0.002458656
SOCS3	9021	Suppressor of cytokine signaling 3	−0.006167891
JUN	3725	v-jun avian sarcoma virus 17 oncogene homolog	−0.00134346
ROBO3	64221	Roundabout, axon guidance receptor, homolog 3	0.273163929
IRF7	3665	Interferon regulatory factor 7	−0.006211324
IL18	3606	Interleukin 18	−0.004675985
TNFSF4	7292	Tumor necrosis factor ligand superfamily, member 4	0.108822803
CCR7	1236	Chemokinereceptor-7	0.018881849
CCL24	6369	Chemokine (C-C motif) ligand 24	0.084982098
VAV3	10451	VAV3 guanine nucleotide exchange factor	−0.003920753
NFKBIE	4794	NFKB inhibitor epsilon	−0.03103477
FGF7	2252	Fibroblast growth factor 7	0.088672495
NRG1	3084	Neuregulin 1	−0.26605368
SCG2	7857	Secretogranin 2	0.002140649
ADRB1	153	Adrenoceptor beta 1	−0.093132018
FLT3	2322	Fms-like tyrosine kinase 3	−0.066140388
IL2RG	3561	Interleukin 2 receptor, gamma	−0.002806802
NPR3	4883	Natriuretic peptide receptor 3	0.007104248
SDC1	6382	Syndecan 1	0.000256454
SSTR1	6751	Somatostatin receptor 1	0.041379722
TNFRSF8	943	Tumor necrosis factor receptor superfamily, member 8	−0.463191784

**Figure 3:**
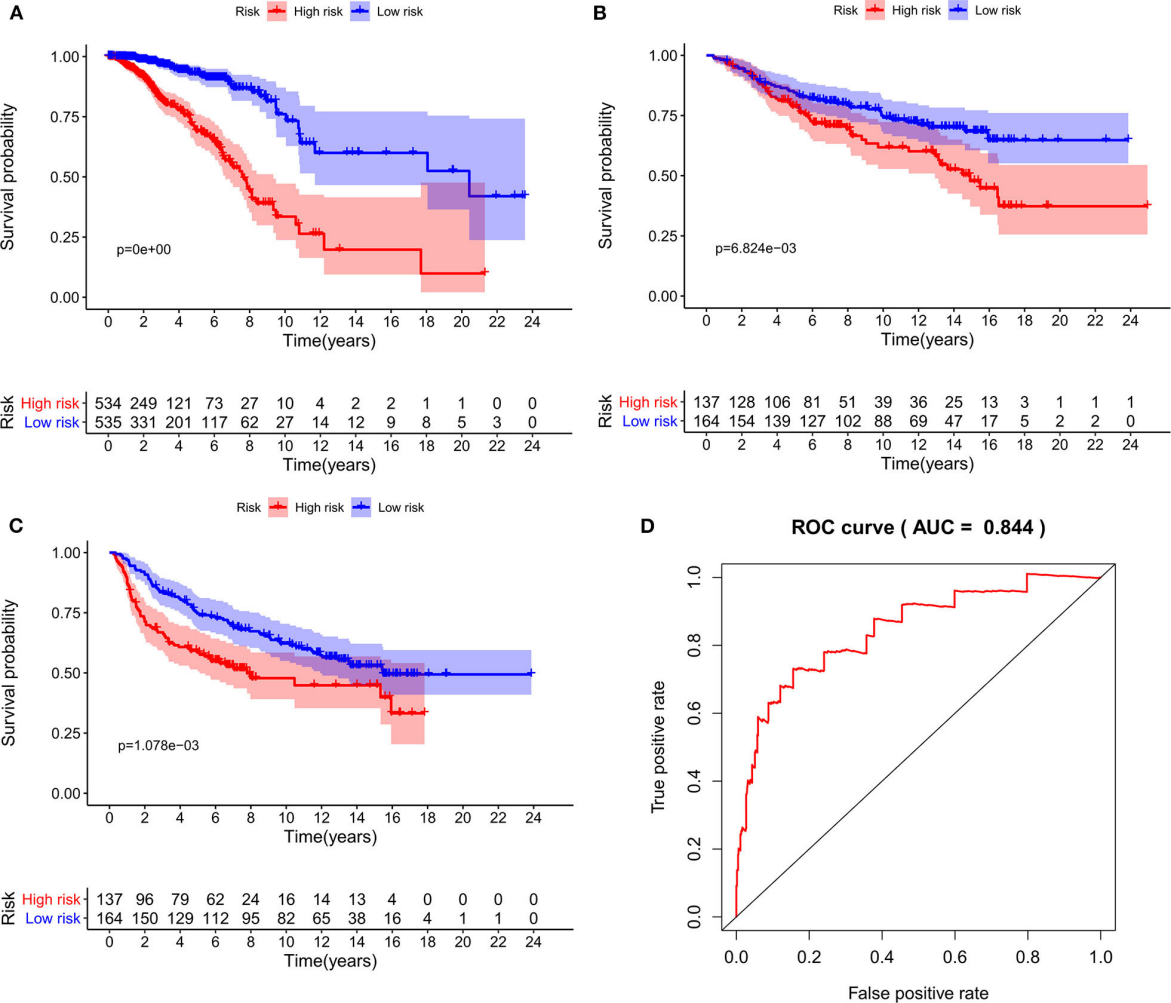
Kaplan-Meier analysis of patients' OS in the high-risk and low-risk subgroups of the training cohort **(A)** and validation cohort **(B)**, Kaplan-Meier analysis of patients' RFS in the high-risk and low-risk subgroups of the validation cohort **(C)**, the x axis represents the survival time (years), and the Y axis represents survival rate. The AUC of ROC curve was 0.844 showing the superior predictive accuracy of survival **(D)**.

**Figure 4:**
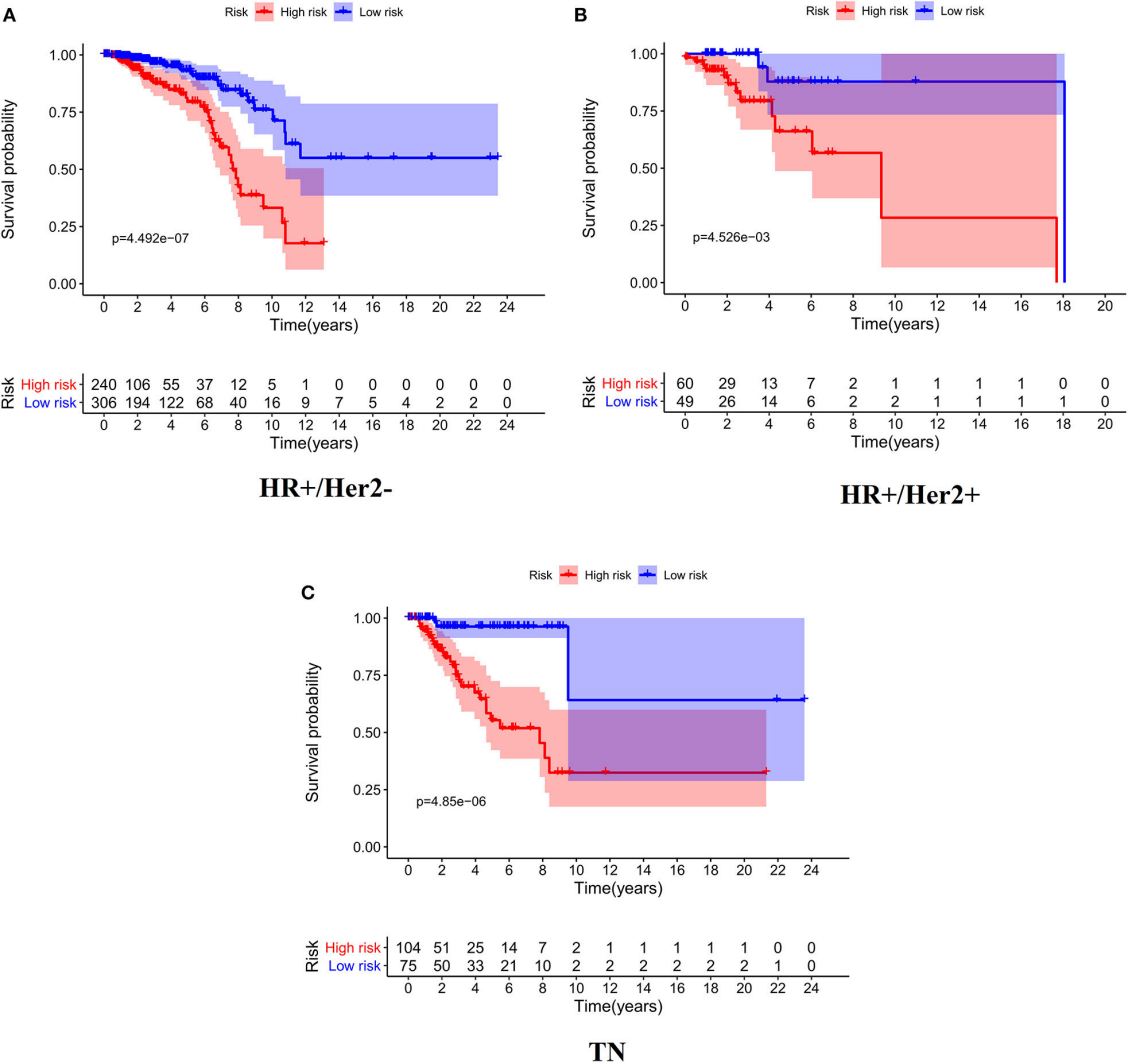
Kaplan-Meier analysis of patients' OS in the high-risk and low-risk subgroups of the HR+/Her2– **(A)**, HR+/Her2+ **(B)**, and TN **(C)** BC.

As expected, low-risk BC patients exhibited significantly longer OS relative to high-risk BC patients (16.73 ± 1.066 vs. 8.963 ± 0.797 years, *p* < 0.0001) (Figure 3), and the prognostic accuracy of this model was further supported by a time-dependent ROC analysis that yielded an AUC value of 0.844. We then sought to validate this IRGS risk score by applying the same formula and cut-off values to our validation dataset, in which we similarly observed longer survival in low-risk patients relative to high-risk patients (17.965 ± 0.756 vs. 14.727 ± 1.005 years; *p* = 0.0006). We additionally found that recurrence-free survival (RFS) in low-risk patients was also longer than in high-risk patients (15.071 ± 0.778 vs. 9.511 ± 0.705 years; *p* = 0.0001). Moreover, the prognostic impact of IRGS-derived risk scores was evaluated in different molecular subtypes of BC. Similarly, patients in the low-risk group showed longer OS in HR+/Her-2- (16.671 ± 1.362 vs. 7.983 ± 0.503 years, *p* < 0.0001), HR+/Her-2+ (16.318 ± 1.418 vs. 9.137 ± 2.114 years, *p* < 0.0001), and triple negative BC (18.242 ± 3.711 vs. 9.938 ± 1.55 years, *p* < 0.0001) (Figure 4).

In a univariate analysis, we detected a significant relationship between IRGS scores and BC patient OS in both the training cohort (HR = 3.608, 95% CI 2.927–4.447, *p* < 0.001, Table 3) and the validation cohort (HR = 1.725, 95% CI 1.157–2.574, *p* = 0.008). A multivariate analysis similarly confirmed that IRGS risk scores were independently associated with patient OS in both the training cohort (HR = 3.315, 95% CI 2.653–4.143, *p* < 0.001) and the validation cohort (HR = 1.523, 95% CI 1.006–2.305, *p* = 0.047).

**Table 3:** Univariate and multivariate analysis of IRGS and clinical factors of BC patients in training and validation cohort.

**Characteristic**	**Univariate**		**Multivariate**	
	**HR (95% CI)**	***p*-value**	**HR (95% CI)**	***p*-value**
**Training cohort**				
Age	1.035 (1.021–1.05)	<0.001	1.035 (1.02–1.05)	<0.001
Gender	0.879 (0.123–6.303)	0.993	0.26 (0.033–2.08)	0.204
T stage	1.577 (1.262–1.971)	<0.001	0.947 (0.703–1.277)	0.723
N stage	1.655 (1.372–1.997)	<0.001	1.319 (0.96–1.81)	0.087
M stage	6.695 (3.666–12.225)	<0.001	1.444 (0.653–3.375)	0.397
TNM stage	2.236 (1.75–2.858)	<0.001	1.427 (0.825–2.467)	0.203
ER (-)	0.673 (0.454–0.996)	0.047	0.912 (0.493–1.69)	0.771
PR (-)	0.677 (0.468–0.978)	0.038	0.714 (0.402–1.269)	0.251
Her-2 (-)	1.189 (0.747–1.891)	0.466	0.86 (0.534–1.384)	0.534
IRGS	3.608 (2.927–4.447)	<0.001	3.315 (2.653–4.143)	<0.001
**Validation cohort**				
Age	2.111 (1.208–3.692)	0.009	1.31 (0.72–2.382)	0.377
T stage	1.387 (1.177–1.635)	<0.001	1.165 (0.976–1.39)	0.091
N stage	3.941 (2.443–6.358)	<0.001	2.68 (1.543–4.655)	<0.001
Grade	1.603 (1.177–2.182)	0.003	1.463 (1.062–2.015)	0.02
IRGS	1.725 (1.157–2.574)	0.008	1.523 (1.006–2.305)	0.047

### Functional Enrichment Analysis

**Figure 5:**
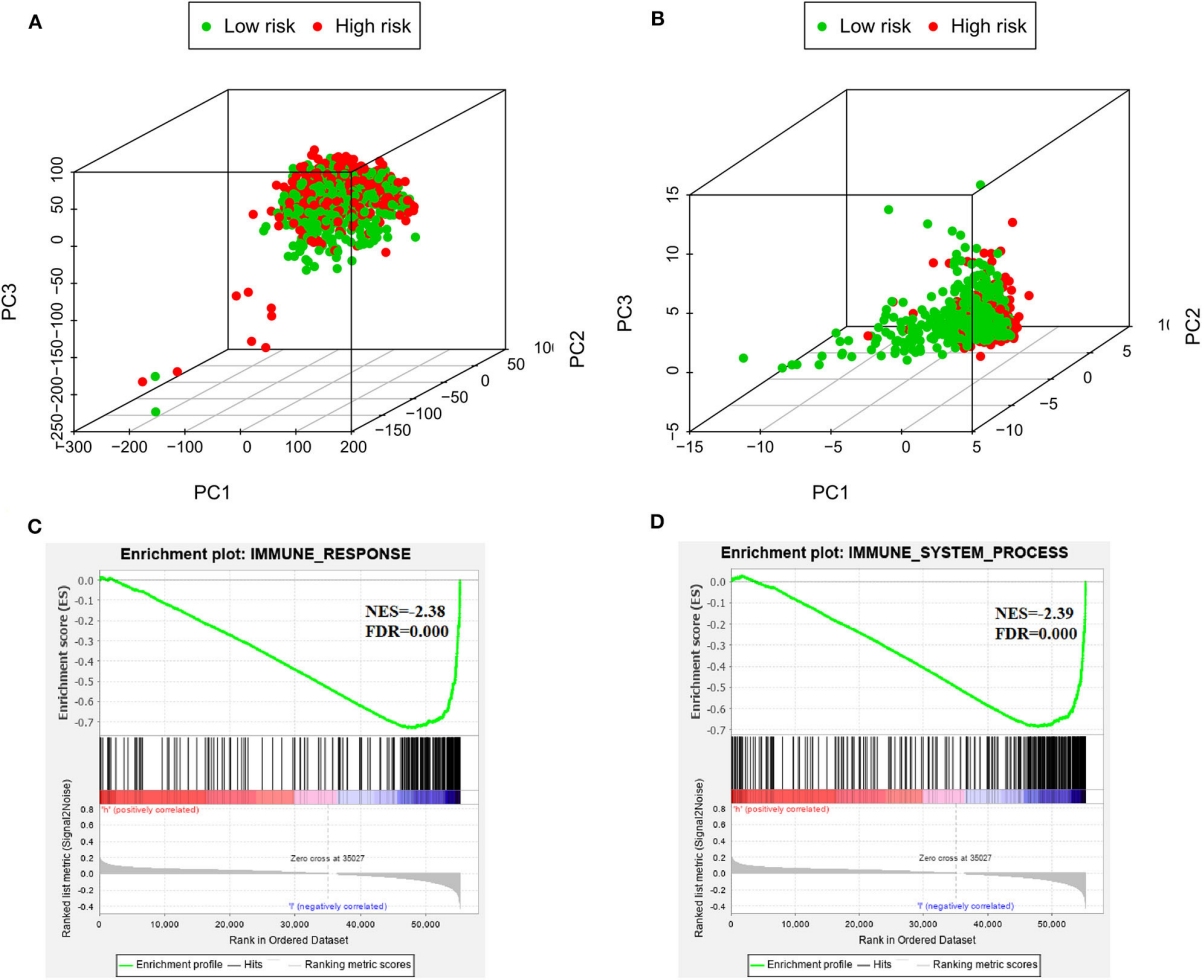
The low-risk and high-risk groups displayed different immune statuses. Principal components analysis between low- and high-risk groups on the basis of the whole genome expression data **(A)** and the immune-related gene signature **(B)**. Functional annotation of the IRGS by GSEA analysis: **(C)** Immune response, **(D)** Immune system process.

PCA was used to investigate the different distribution patterns between low- and high-risk groups on the basis of the IRGS and whole gene expression profiles, the low- and high-risk groups tended to separate into two sides in IRGS set (Figures 5A,B). We additionally employed a GSEA functional annotation approach which revealed that low-risk patient samples were significantly enriched for genes associated with immune response and immune system processes relative to samples from high-risk patients (Figures 5C,D).

**Figure 6:**
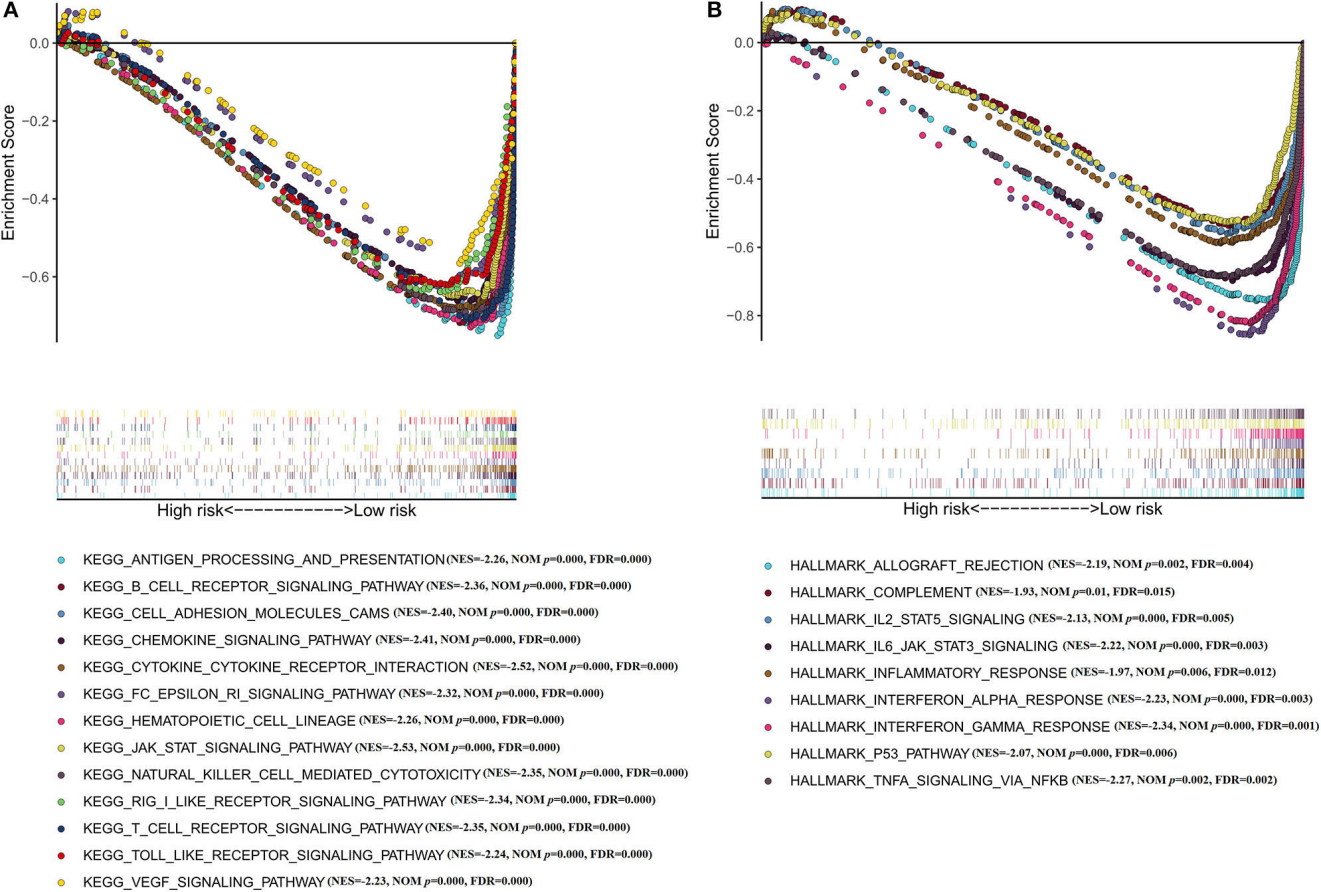
Functional annotation of the IRGS by GSEA analysis **(A)** KEGG; **(B)** Cancer hallmarks.

Moreover, GSEA has been implicated in KEGG (Figure 6A) and cancer hallmarks (Figure 6B). The enriched cancer hallmarks in low-risk group, including the interferon alpha (IFN-α) response, the interferon-γ (IFN-γ) response, IL-2/STAT5 signaling, IL-6/JAK/STAT3 signaling, epithelial mesenchymal transition, TGF-β signaling and hedgehog signaling. Besides, the enriched pathways for low-risk group were predominantly involved in immune-related pathways, including JAK-STAT signaling, VEGF signaling, Toll-like receptor signaling, RIG-I-like receptor signaling, B cell receptor signaling, T cell receptor signaling, natural killer cell-mediated cytotoxicity, antigen processing and presentation, hematopoietic cell lineage and others.

### Clear Differences in Immune Infiltration Are Evident When Comparing Low- and High-Risk BC Patients

**Figure 7:**
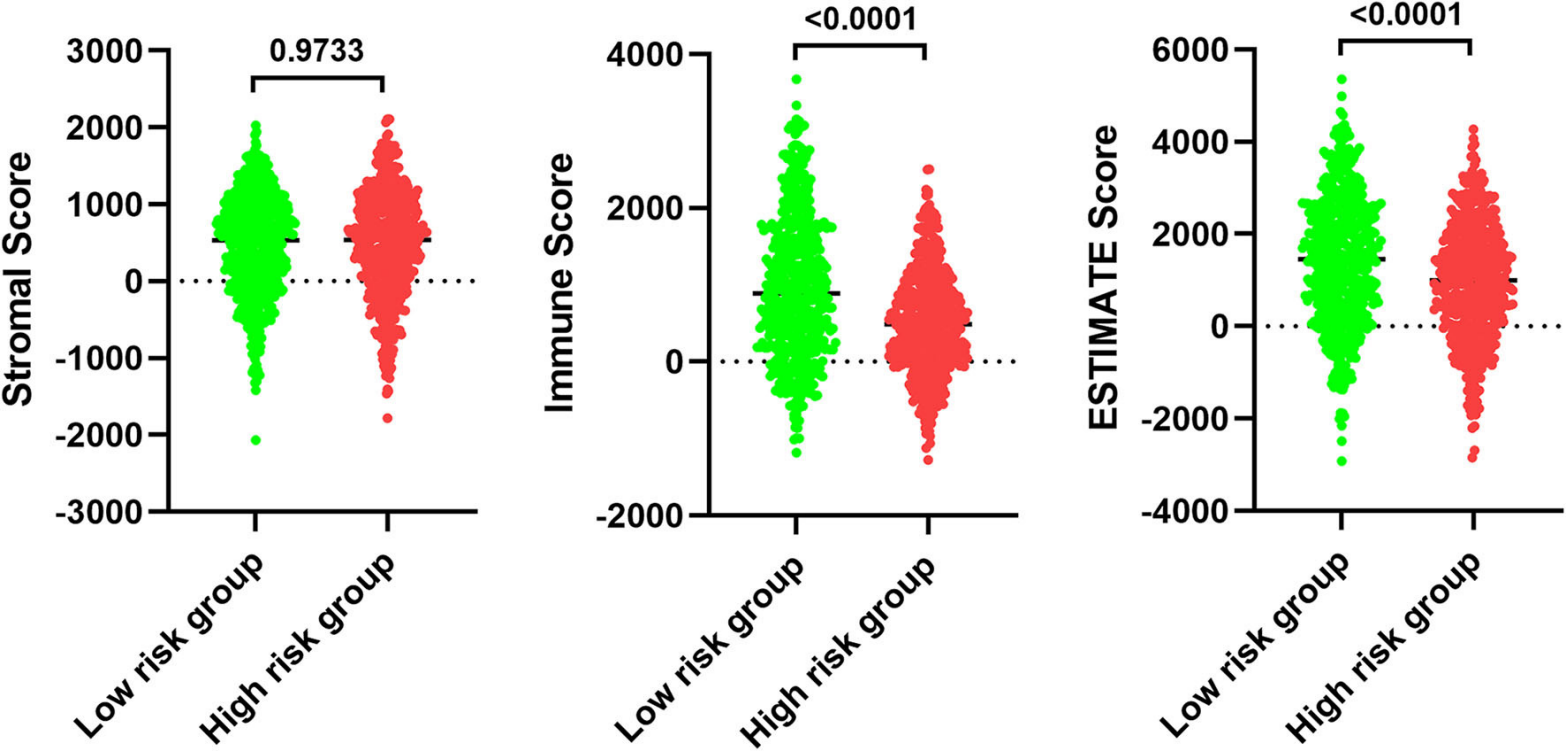
Analysis of ESTIMATE algorithm to the TCGA dataset.

**Figure 8:**
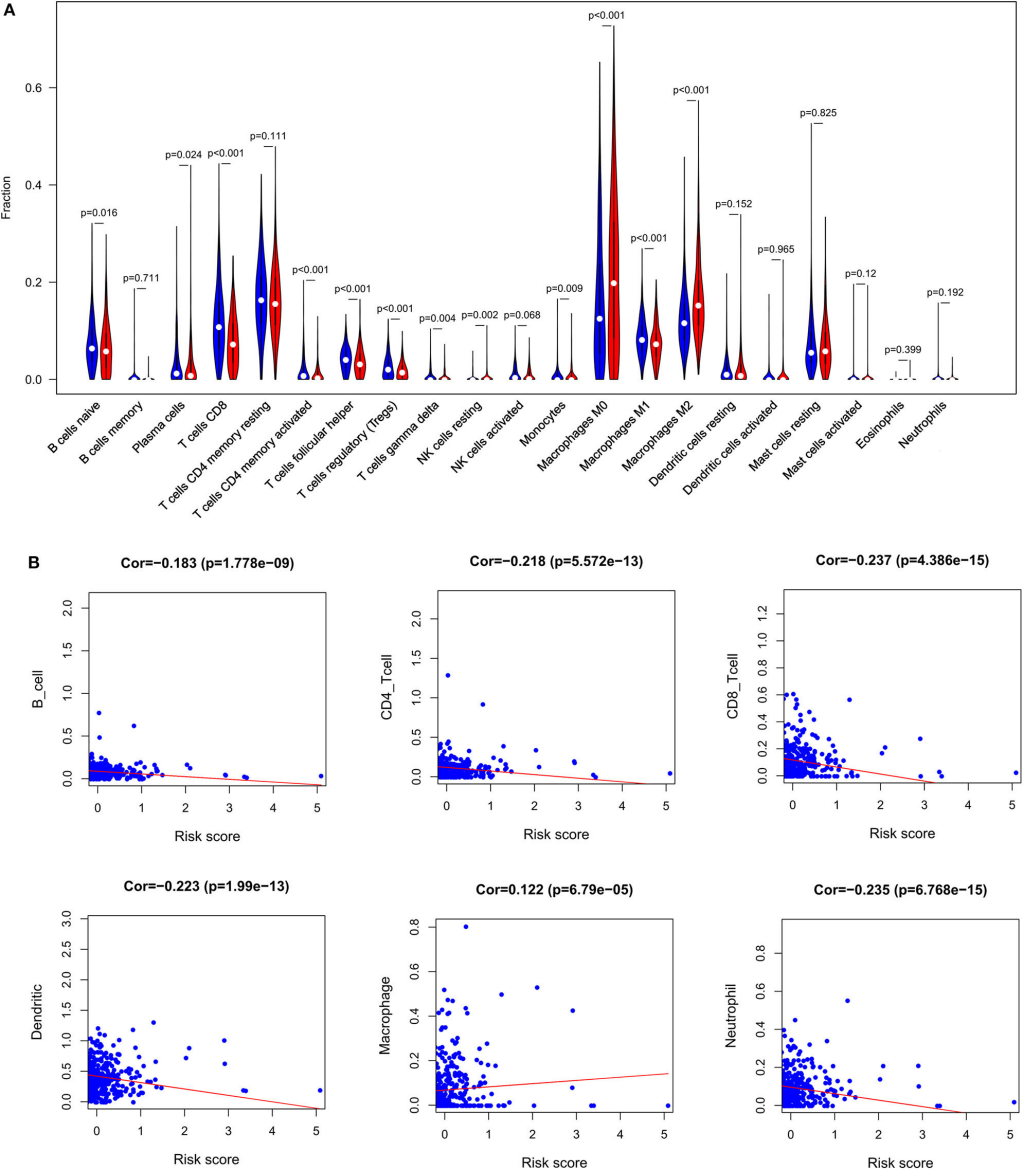
Immune analysis. **(A)** The violin plot showed the immune cells infiltration in the low- and high-risk groups. **(B)** Correlation analysis between immune cells and risk score.

**Figure 9:**
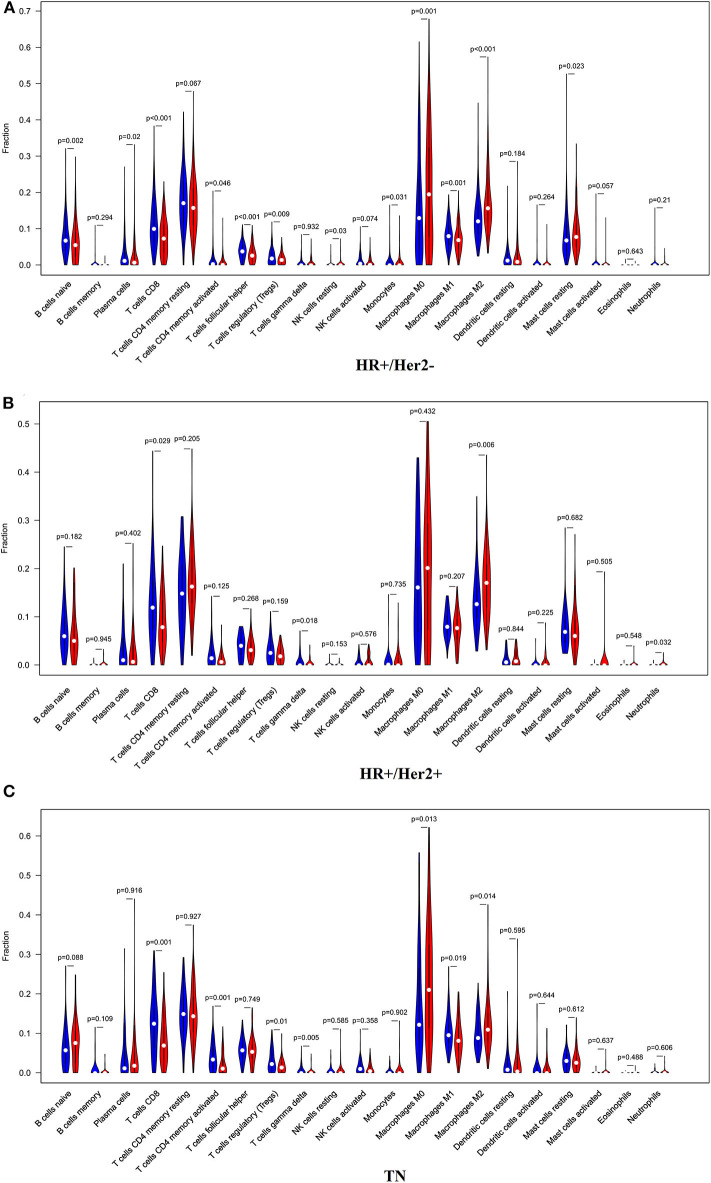
The violin plot showed the immune cells infiltration in the low- and high-risk groups of the HR+/Her2– **(A)**, HR+/Her2+ **(B)**, and TN **(C)** BC.

In order to examine the relative contributions of stromal and immune cells in these BC samples, we utilized the ESTIMATE algorithm in order to process these TCGA data. This analysis revealed that high-risk BC patients exhibited a significantly lower degree of immune infiltration relative to low-risk patients, with immune scores differing significantly between these groups (*p* < 0.0001) even though stromal scores did not (*p* = 0.8785) (Figure 7). When we conducted an immune cell type-specific analysis, we found that low-risk patients exhibited higher levels of naive B cells, plasma cells, CD8 T cells, CD4 memory activated T cells, follicular helper T cells, regulatory T cells (Tregs), γδ T cells, resting NK cells, monocytes, and M1 macrophages, whereas high-risk patients exhibited higher levels of M0 and M2 macrophages (Figure 8A). Correlation analyses revealed IRGS risk scores to be significantly negatively correlated with B cell, CD4+ T cell, CD8+ T cell, dendritic cell, and neutrophil enrichment, and to be positively correlated with macrophage enrichment (Figure 8B). Besides, the immune cell type-specific analysis was conducted in different molecular subtypes of BC, we found that low-risk patients exhibited higher levels of CD8+ T cells and lower levels of M2 macrophages in all subtypes of BC (Figure 9).

**Figure 10:**
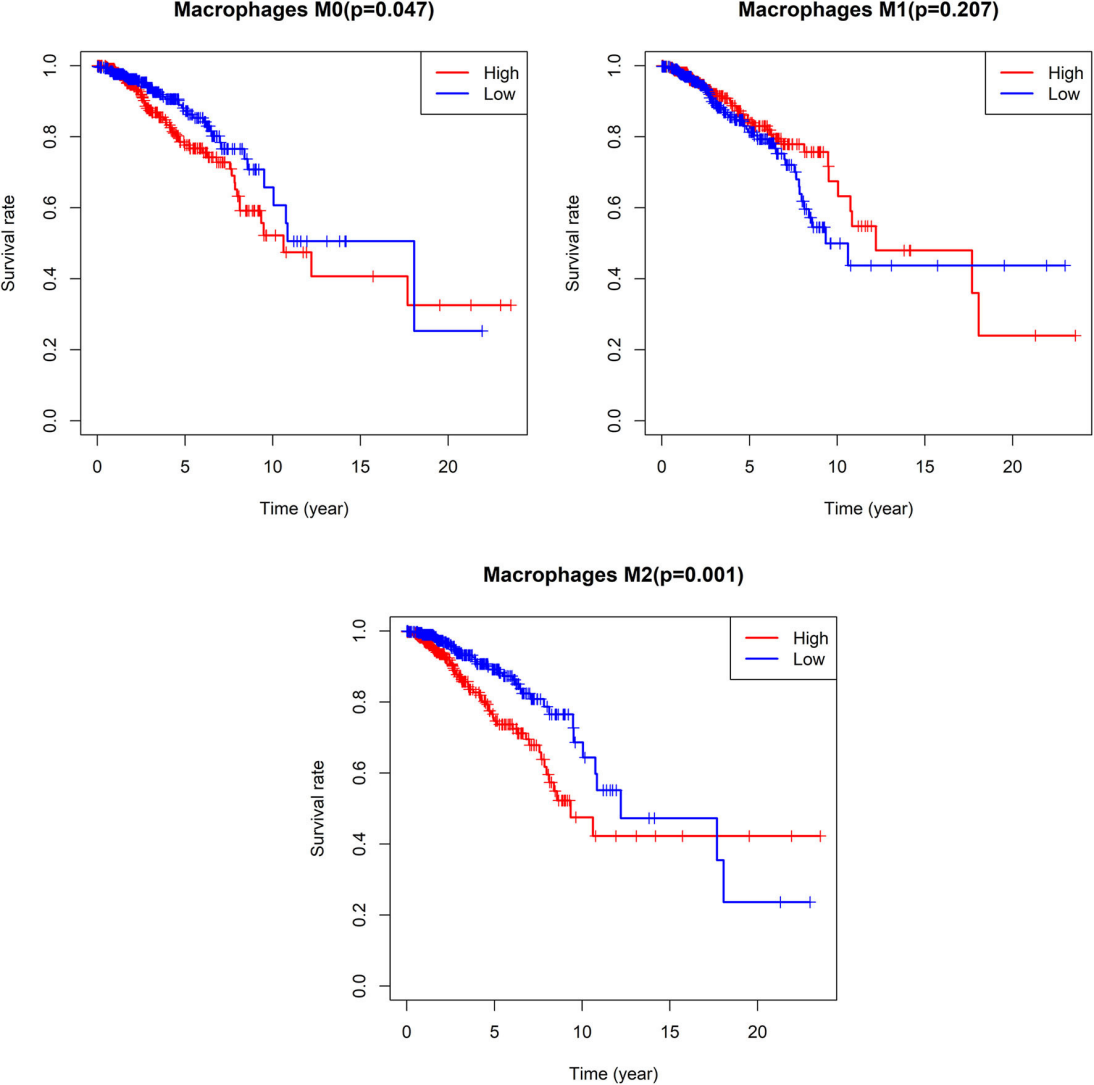
Survival analysis according the cell content of M0-macrophages, M1-macrophages, and M2-macrophages. The cut-off point between high and low groups is median-value.

We additionally found that patients with lower M0 and M2 macrophage levels exhibited significantly longer OS on average when compared to patients with higher levels of these cells (M0: median OS 18.063 vs. 10.611 years, *p* = 0.047; M2: median OS 12.208 vs. 9.34 years, *p* = 0.001), whereas no such relationship was detected for M1 macrophages (Figure 10).

## Discussion

Previous research strongly suggests that immune cell infiltration of tumors can strongly impact patient clinical outcomes in many different cancer types including BC, ovarian cancer, and melanoma (11–13). While difficult to assess directly in many cases, such immune infiltration can be effectively gauged through analyses of gene expression datasets from patient tumor samples. In the present study, we began by leveraging TCGA and GEO BC patient datasets in order to identify BC-related immune-related genes. We then examined the relationship between gene expression patterns and BC patient clinical data in order to identify survival-associated DEIGs, and generated an IRGS risk model that could be used to effectively stratify BC patients into low- and high-risk groups exhibiting significant differences in survival, immune cell infiltration of tumors, and immune/stromal content.

The immune system is an essential mediator of clinical outcomes in solid tumor patients. We were able to identify 38 total DEIGs associated with BC patient survival outcomes, and we then used these DEIGs to construct our IRGS risk model. IRGS risk scores allowed us to successfully stratify BC patients into low- and high-risk groups with significant differences in both OS and RFS, as well as a high AUC value. Recently, a number of studies exploring the prognostic immune-genes for BC patients were reported. Bai et al. (14) identified DEGs in BC samples with high and low lymphocyte-specific kinase (LCK) metagene scores. They obtained 115 genes, and found that 22 of them were independent predictors of OS in BC patients. Ren et al. (15) screened 248 intersecting DEGs in stromal score differential genes and immune score differential genes, they identified 31 prognosis-related genes from the tumor microenvironment for BC patients. However, we utilized lists of immune-related genes from the ImmPort to identified DE immune-related genes. To the best of our knowledge, it is the first study to focus on IRGS associated with BC patient survival. We also evaluated the relevance of this signature to immune response and immune cell infiltration of BC.

Among these 27 genes enrolled in IRGS, some have previously shown to correlate with the tumorigenesis of BC. For example, suppressor of cytokine signaling 3 (SOCS3), as an important negative regulator of JAK/STAT pathways, serve as essential signaling transduction intermediaries in response to a number of different cytokines and immune responses in BC (16–18). GSEA revealed that low-risk patient samples exhibited gene expression patterns enriched for genes associated with the immune system, while high-risk patients had lower levels of immune cell infiltration. This may suggest that enhanced immune infiltration is associated with better outcomes in BC patients and that the further study of IRGS-related pathways may highlight novel approaches to extending the life expectancy of BC patients.

The TME is a current topic of intense scientific interest. Different types of tumor-infiltrating lymphocytes (TILs) have been found to be associated with significant differences in BC patient outcomes, with T cells being the best studied and understood of these TILs. T cells are typically the most abundant immune cell type within BC tumors, and recent work indicates that CD8+ T cells play a key role in preventing BC metastasis and in shaping overall patient outcomes (19–22). Consistent with this, we found that CD8+ T cells were enriched in low-risk patients and were negatively correlated with patient risk scores. Tumor-infiltrating regulatory T cells (Tregs) have also been heavily studied in the context of BC, although these previous studies have yielded inconsistent findings (23–26). Some reports suggest that Tregs are associated with poor patient prognosis, whereas others suggest the opposite. In a meta-analysis conducted by Shang et al. (27), Tregs infiltration was shown to be associated with favorable outcomes in ER– BC, but with a poor prognosis in ER+ BC patients, potentially explaining these discrepancies. In this study, we found that Tregs were enriched in tumor samples from low-risk patients. The relevance of B cell infiltration to BC patient outcomes is not as well-understood, and the role of these cells thus remains controversial in this context (28, 29). In the present study, we found B cell infiltration to be significantly negatively correlated with BC patient risk scores, and we observed significant enrichment of naive B/plasma cells in low-risk patients. Future studies, however, will be required in order to understand the exact prognostic relevance of these cells.

Tumor-associated macrophages (TAMs) have also been thoroughly studied as key mediators of interactions between tumors and the immune system (30). These TAMS are generally grouped into two major subtypes: M1 and M2 macrophages. M1 macrophages secrete inflammatory factors including TNF-α and IL-12, and typically suppress tumor development. In contrast, M2 macrophages express immunosuppressive cytokines and growth factors like IL-10, Arg-1, CD206, VEGF, and EGF, and can thereby promote tumor proliferation, metastasis, and angiogenesis (31). Several prior studies have demonstrated that such M2 macrophage infiltration is associated with poorer anti-tumor immune response, with increased ECM remodeling, and with enhanced angiogenesis (32). In the present study, we found that high-risk patient samples were enriched for M2 macrophages, while patients with low levels of M2 cells exhibited a significantly longer OS relative to patients with high levels of these cells. The specific factors influencing M1- and M2-macrophage polarization in the BC tumors of these patients are not clear. JAK/STAT signaling is well-known to be a key regulator of such polarization (33), and our GSEA results indicated that low-risk patients were enriched for IFN-γ responses, IL-6/JAK/STAT3 signaling, and IL-2/STAT5 signaling. IFN-γ can activate STAT1, thereby promoting M1 polarization via the IFN-γ/JAK/STAT1 signaling pathway (34). In contrast, IL-6/JAK/STAT3 signaling has largely been suggested to promote M2 macrophage polarization (35, 36). Besides, many other factors can influence macrophage fate determination including SOCS1, SOCS3, and hsa-miR-155 (37). Indeed, reductions in SOCS3 expression and enhanced STAT3 activation can promote M2 macrophage polarization. In addition, the inhibition of miR-155/SOCS1 expression by AKT1 can similarly support the development of M2 macrophages (38).

This study has multiple limitations. For one, this was a retrospective analysis, and future prospective studies are therefore required. Furthermore, additional *in vitro* and *in vivo* functional analyses will be needed to validate and expand upon these results. In addition, while we made efforts to minimize cross-study batch effects in our analyses, such effects cannot be completely eliminated. Lastly, while we were able to attempt to reconstruct the intratumoral immune landscape based on our gene expression data, it is important to remember that such cellular composition is not necessarily reflective of true cell-cell interactions within these tumors. Further validation of all aspects of results is therefore required.

## Conclusion

Together, our results offer a novel approach to comprehensively synthesizing immunological genes data in order to reliably gauge BC patient prognosis. These findings may allow us to identify previously unidentified molecular targets amenable to immunotherapy-mediated treatment, thereby improving clinical options for BC patients. However, future studies will be required in order to validate the clinical utility and prognostic relevance of the IRGS described in this study.

## Data Availability Statement

The datasets generated for this study can be found in The Cancer Genome Atlas (TCGA; *n* = 1,222, 113 normal breast samples and 1109 BC samples) and separate gene expression omnibus (GEO) microarray datasets (GSE42568, 104 BC samples; GSE7390, 198 BC samples).

## Ethics Statement

Ethical review and approval was not required for the study on human participants in accordance with the local legislation and institutional requirements. Written informed consent for participation was not required for this study in accordance with the national legislation and the institutional requirements.

## Author Contributions

YZ performed the data analyses and wrote the manuscript. CP helped to perform the data analysis. ZL contributed to data analysis and editing of the manuscript. All authors contributed to the article and approved the submitted version.

## Conflict of Interest

The authors declare that the research was conducted in the absence of any commercial or financial relationships that could be construed as a potential conflict of interest.
